# A Preliminary Study on the Relationship between Platelet Serotonin Transporter Functionality, Depression, and Fatigue in Patients with Untreated Chronic Hepatitis C

**DOI:** 10.1155/2014/821381

**Published:** 2014-03-20

**Authors:** Leonora Franke, Eric Therstappen, Beate Schlosser, Michael Biermer, Thomas Berg, Martin Schäfer, Petra Arck, Ralf Uebelhack, Astrid Friebe

**Affiliations:** ^1^Charité University Medicine Berlin, CCM, Department of Psychiatry and Psychotherapy, 10117 Berlin, Germany; ^2^Charité University Medicine Berlin, CCM, Department of Medicine, Division of Psychosomatic Medicine and Psychotherapy, 10117 Berlin, Germany; ^3^Augusta Victoria Hospital, Department of Infectiology and Gastroenterology and Hepatology, Berlin, Germany; ^4^Leber- und Studienzentrum am Checkpoint Berlin, Germany; ^5^University Clinic Leipzig, Department of Hepatology, Clinic of Gastroenterology and Rheumatology, Leipzig, Germany; ^6^Department of Psychiatry and Psychotherapy, Kliniken Essen-Mitte, Essen, Germany; ^7^University Hospital Hamburg-Eppendorf, Laboratory for Fetomaternal Medicine, Department of Obstetrics and Fetal Medicine, Hamburg, Germany; ^8^Ruhr University Bochum, Department of Psychiatry, LWL University Hospital, Bochum, Germany

## Abstract

*Objective and Methods.* Although the interaction between fatigue and depression in patients with chronic hepatitis C infection (HCV) has been recognized, the biological correlates of this observation have yet to be reported. We addressed this issue by examining serotonin transporter- (SERT-) driven [^14^C]-serotonin uptake rate (SUR) and serotonin content in platelets of 65 untreated HCV patients and 65 healthy control subjects (HCS). All patients completed report questionnaires for fatigue, depression, and general psychopathology. Structured interviews were conducted by a board-certified psychiatrist. * Results.* Whereas 36 of the patients experienced fatigue of moderate-to-severe intensity, only 16 reported symptoms of depression (BDI score > 10). Mean SUR in patients with depressive symptoms was significantly higher relative to the HCS, corresponding to a large Cohen's effect size of *d* = 1.45 (95% CI = 0.66—1.83). Patients who rated their fatigue to have a marked impact on mood and activity displayed a moderate relationship between the BDI score and SUR (*n* = 18, *r* = 0.563, *P* = 0.015), which becomes stronger after controlling for age, gender, and thrombocytopenia (*r*_part_ = 0.710, *P* = 0.003). In the univariate analysis, high fatigue interference score, thrombocytopenia, and high SUR were all significant predictors of depression. * Conclusions.* High SERT activity could be implicated in the expression of depressive symptoms especially in a subgroup of HCV patients who are feeling fatigue as markedly distressing.

## 1. Introduction

One of the primary liver diseases with significant psychiatric comorbidity is hepatitis C virus infection (HCV). Given the high comorbidity of affective disorders and substance abuse, it is not surprising that in an early study on 309 drug users with and without HCV, significant depression symptoms were present in 57.2% and 48.2%, respectively [1]. However, the aetiology of depression among HCV-infected patients is not completely understood. One hypothesis proposed that the HCV-stimulated immunological and inflammatory response is important not only for the development of liver injury but also causes psychiatric symptoms [2].

Depression associated with untreated chronic HCV has been reported to occur in 6% to 30% of patients, with differences in reported rates affected by choice of diagnostic criteria, screening tools, and study population [3–6]. The psychiatric evaluation of patients with HCV was commonly performed by clinicians with limited psychiatric training. Instead of an in-person interview with trained psychiatrists, the diagnosis of “depression” was based on standardized self-report questionnaires.

One of the most common and disabling symptoms experienced by most but not all patients with chronic HCV is fatigue [7]. The relationship between fatigue and depression is not frequently investigated in HCV patients, but many hepatologists believe that the presence of severe fatigue is associated with depression. Dwight et al. (2000) reported that severity of depression symptoms, expressed as the Beck Depression Inventory score and a global fatigue score, correlated significantly in a group of 50 US-American patients with chronic HCV [4]. Because fatigue was found not to be affected by liver disease severity, medical comorbidity, or demographic variables, the authors concluded that depression has a more significant impact on fatigue and functional impairment than the severity of liver disease. Whether it is the presence of symptoms of depression that contributes to fatigue, or vice versa, has yet to be established. McDonald et al. (2002) examined 115 chronic HCV patients and found that the psychopathology domains of the SCL-90-R --, particularly depression, anxiety, and interpersonal sensitivity --, strongly correlated with scores on the Fatigue Impact Scale [8].

Based on the assumption that the association between severe fatigue and depressed mood in HCV patients exists specifically in patients with disturbed serotonin signaling, our study explored the possible alteration in serotonin transporter (SERT) functionality in platelets of nontreated HCV patients. In the past, platelet SERT and 5HT concentration have been studied extensively as an indirect index of central serotonergic function in a variety of psychiatric and neurological disorders [9–13]. With regard to patients with HCV, the few studies that analyzed changes in whole blood, serum, or platelet-5HT concentration in the course of interferon-*α* (IFN-*α*) treatment showed a significant and gradual decrease of 5HT with treatment duration [14–18]. To our knowledge, there are no studies that have compared platelet 5HT content and/or SERT activity in nontreated HCV patients and healthy subjects.

The SERT plays a critical role in regulating the intensity and duration of serotonergic signalling [19]. It is notable that proinflammatory cytokines IL-1, IFN-*α*, IFN-*γ*, and TNF-*α* have been shown to increase the activity and expression of the SERT* in vitro* and* in vivo* [20–22]. In contrast, the anti-inflammatory cytokine, interleukin-4, seems to induce a dose-dependent reduction in 5HT uptake activity in immortalized B lymphocytes [23].

Based on these observations, we assumed that the SERT-driven uptake of 5HT in platelets would be high, at least in subsets of patients with chronic HCV. High SERT activity should be associated with high 5HT content in platelets, provided that the gastrointestinal 5HT synthesis is not disturbed. The present study examined whether untreated patients with HCV are different from healthy control subjects with respect to these platelet indices and whether the possible abnormalities are specifically related to patients who exhibit symptoms of depressive disorders. We included different measures of fatigue in our data analysis in an effort to better understand the nature of depressive symptoms in HCV patients. Here we report results of a prospective study which assembled numerous baseline data of 65 patients with chronic HCV who underwent evaluation for antiviral treatment.

## 2. Material and Methods

The study protocol conforms to the ethical guides of the 1975 Declaration of Helsinki and was approved by the Ethical Committee of the Charité University Hospital. The patients underwent evaluation and management at the outpatient clinic at the Division of Hepatology, Gastroenterology, and Endocrinology, Campus Virchow-Klinikum, Charité University Medicine Berlin from May 2007 to April 2009. Written informed consent was obtained from patients prior to an in-person psychiatric interview.

We analysed baseline data on 65 nontreated patients with clinical diagnosis of chronic HCV (29 males/36 females, aged 21–72 years) as a part of a study examining psychiatric symptoms as well as immune and neurobiological variables prior to and during the treatment with IFN-*α*/ribavirin.

Exclusion criteria for our study included decompensated liver disease, active use of illicit injection drugs, ongoing excessive alcohol consumption, coinfection (hepatitis B virus or HIV), other autoimmune disorders, immunomodulating medication, or other severe internal diseases. Patients were required to be off all antidepressant, antipsychotic, or mood stabilizing medication for at least 3 months and to be abstinent from illicit drugs/alcohol for at least 6 months prior to entering the study.

The control group of healthy subjects (*n* = 65) was matched for age (mean age 46.8, range 21–69 years), gender (29 males/36 females), BMI (mean BMI 25.1 kg/m², range 18.5–30.3), and smoking status (32.3% were smokers and smoked 2–20 cigarettes per day). Hypertension was documented in 12 subjects aged between 46 and 69 years. Control subjects had no medical history of any psychiatric disorder. About one-half of healthy control subjects (HCS) completed the Brief Symptom Inventory.

All patients received a semistructured diagnostic interview by the same board-certified psychiatrist using the Mini International Neuropsychiatric Interview (MINI). The MINI (German version 5.00) is a diagnostic instrument for making DSM-IV and ICD-10-validated categorical diagnosis of the major Axis I psychiatric disorders [24]. Six patients (five males and one female, aged 46–68 years) met the diagnostic criteria for major depression: a symptom count greater than or equal to five of the nine symptoms specified for depression with a duration greater than or equal to two weeks. In addition, minor depression was diagnosed in six patients (four males and two females, aged 23–52 years). Minor depression was defined as having three or four of the nine depression symptoms, with duration of two weeks or more. The psychiatrist was blinded to the BDI and Brief Symptom Inventory score at the time of interview.

## 3. Assessments

All patients' baseline laboratory tests, including complete blood counts, metabolic panel, liver, and thyroid function tests, were collected from their medical record.

To quantify the symptoms of depression as well as general psychopathological symptoms, patients completed three measures.

The* Brief Symptom Inventory (BSI)* is the short form of the Symptom Check List-90-Revised. This 53-item inventory measures the presence and severity of behaviour problems and psychopathological symptoms. Responders were asked to report how they had felt during the previous 7 days on a 4-point scale. Following standard procedure for the BSI, raw scores were transformed to *T*-scores with a normative population mean of 50 and a standard deviation of 10. For group comparisons, the subscales “depression,” “anxiety,” “anger-hostility,” and the “general severity index” (GSI) were included.

The* Beck Depression Inventory (BDI)* was originally designed to measure the presence and intensity of typical symptoms of depression in patients who have already been diagnosed by a psychiatrist according to standard criteria. The BDI consists of 21 items, each scored from 0 to 3.

The BDI is thought to be an adequate tool for ruling out clinically significant depressive disorders in different HCV populations [25–27]. We applied the conventional cutoffs for classification of BDI scores into nondepressed (≤10) and depressed (>10).

The* Brief Fatigue Inventory (BFI)* was originally developed to measure the intensity of fatigue and how much fatigue interferes with physical, cognitive, and psychosocial activities in cancer populations. It consists of nine items measured on a 0–10 numeric rating scale. For three of the items, patients are asked to rate the intensity of their fatigue (weariness, tiredness) during the past 24 hours: fatigue at present, at its usual level, and at its worst. The scale applied to these items defines 0 as “no fatigue” and 10 as “fatigue as bad as you can imagine.” Six items assess how much fatigue interferes with different aspects of their daily activities during the past 24 hours, including general activity, mood, walking ability, normal work, relations with others, and enjoyment of life, with 0 being “does not interfere” and 10 being “completely interferes.”

Because the nine items of the BFI load into a single dimension, the arithmetic mean of the nine items is interpreted as severity of fatigue [28].

Based on methods used for validating the BFI by Mendoza et al. [28, 29], or other validation studies of the BFI, [30], the present study used the following measures for data analysis: (1) global score (arithmetic mean of all nine BFI items); (2) mean fatigue severity score (arithmetic mean of first three BFI items); (3) mean fatigue interference score (arithmetic mean of BFI items 4–9); and (4) categorical fatigue severity based on response to item 3 (fatigue at its worst) only: none (0), mild (1–3), moderate (4–6), and severe (7–10).

The BFI has also been applied in some studies on patients with HCV [31] or with hepatocellular carcinoma [32].

## 4. Blood Collection and Biochemical Assessments

Venous blood samples (9 mL) were collected into vacutainer tubes containing EDTA from a cubital vein between 9:00 and 11:00 a.m. After centrifugation at 250 ×g and 20°C for 15 min, the supernatant containing the platelet-rich plasma (PRP) was collected and the number of platelets in the PRP was counted with a cell counter (MÖLAB, Hilden, Germany). To obtain PRP with appropriate platelet count also in patients with thrombocytopenia, the centrifugation time was reduced to 5-6 min.

## 5. Assay of 5HT Concentration in Platelets

Aliquots of 500 *μ*L PRP were spun at 2000 ×g and 20°C for 5 min. The supernatant was discarded and the platelet pellet was washed once with physiological saline, resuspended in 200 *μ*L saline, and kept at −20°C until assayed. 5HT concentration in platelets was measured by HPLC with UV detection at 220 nm using a reverse-phase cartridge LiChrospher 100 RP18 column as described [33].

The platelet-5HT content was expressed as ng/10^9^ platelets.

## 6. Assay of [^14^C]-5HT Uptake in Platelets

Assays were performed within one hour after PRP preparation according to the procedure described previously [13]. For all study participants, the platelet count in PRP was adjusted to 3 × 10^8^ plts/mL by the addition of homologous platelet-poor plasma. To achieve this platelet count also in patients with strong thrombocytopenia, aliquots of PRP were gently centrifuged, and platelet pellets resuspended in an appropriate plasma volume.

The serotonin uptake rate (SUR) was measured by incubating 200 *μ*L PRP with 200 *μ*L saline and 100 *μ*L [^14^C]-5HT solution (final concentration 35 nM) for 5 min at 37°C. Uptake at 0°C was taken as a control for nonspecific binding of [^14^C]-5HT with the platelets. The uptake was terminated by dilution with 1 mL ice-cold saline and platelets were filtered through Whatman GF/B filters and washed with 2 mL ice-cold saline. Under these conditions of physiologically low substrate concentration, both the number of SERT sites and their affinity to the substrate contributed to the uptake activity. The SUR was expressed as pMol [^14^C]-5HT/10^9^ plts. ×5 min⁡.

## 7. Statistical Analysis

Data were analyzed using the statistical software package SPSS 18.0 (SPSS Institute, Chicago, IL, USA). Descriptive statistics were inspected for normal distribution of variables using the Kolmogorov-Smirnov Test, homogeneity of the variance (Levene's test), and outliers. Outliers with standardized residual greater than 3.0 were removed from the data set for subsequent analysis.

Continuous variables were compared using Student's *t*-test or the Mann-Whitney *U*-test when appropriate. Because of the known robustness of generalized linear models (GLM) against nonnormality of distribution, we employed GLM to compare platelet SUR and 5HT content between groups while also controlling for putative confounders such as age, gender, overweight, smoking, and hypertension. These factors were selected based on their possible association with the investigated platelet measures shown in previous studies. In this model, a conservative measure of significance was used (*P* ≤ 0.01) if the assumption of variance homogeneity in all cells was violated.

As an exploratory analysis, we evaluated associations between psychometric measures or between biological and psychometric measures and calculated Spearman rho correlation coefficients. Where indicated, partial correlation coefficients were determined to control for relevant covariates.

Cross-tabular univariate analysis with Chi-square tests was used to ascertain the relationship between each categorical variable in the study and the likelihood of major/minor depression.

To prove whether our study sample is of adequate size to perform valid statistical analysis, we postulated that one SD above the mean of HCS would be clinically important difference in sample means of 5HT uptake rates (SUR) in patients with CHC and HCS (i.e., high standardized effect size *d* = 1). To detect this difference at *α* = 0.05 and power level of 0.8 requires 17 subjects in each group and 64 subjects in each group to detect a smaller difference with a median effect size *d* = 0.5.

We also report Cohen's *d* effect sizes for pairwise contrast of the difference between diagnostic groups for SUR and platelet-5HT content. Effect sizes *d* with 95% CI were calculated using online clinical research calculators available on the Campbell Collaboration website (http://www.campbellcollaboration.org/resources/ effect_size_input.php).

## 8. Results

### 8.1. Patient Characteristics

The basic demographic and clinical characteristics of patients with chronic HCV are reported in Table 1, including BMI, smoking status, HCV genotype, viral load, white blood cell, lymphocyte and platelet count, and other variables of putative importance (past history of illicit substance and/or alcohol abuse or past presence of depressive symptoms).

Almost two-thirds of the patients sample were >40 years of age and about one-half were overweight (BM > 25 kg/m²). Cigarette smoking (3–30 cigarettes per day) was reported by 28 (43%) of the patients. A subset of eligible patients (*n* = 19, 15M/5F) had a self-reported history of illicit substance and/or alcohol abuse. Last drug use was reported to be 1.5–32 years ago. Patients with history of excessive alcohol drinking (*n* = 6) abstained from alcohol for 12–20 months.

Thrombocytopenia, defined as a platelet count <150.000/*μ*L, was present in 17 (26.1%) patients. Patients with thrombocytopenia were on average slightly older than patients with a normal platelet count (48.8 ± 11.1 versus 45.7 ± 11.7 years); they also had significantly higher mean BMI (27.1 ± 4.3 versus 24.4 ± 2.9 kg/m², *z* = −2.321, *P* = 0.020) and ALT and AST levels (data not shown).

Twenty-six patients had previously undergone treatment with IFN-*α*. Elapsed time between earlier IFN-*α* therapy and study initiation was at least 1 year. Comparing patients without and with IFN-*α* therapy in the past, it was evident that the male therapy-naïve group included significantly more patients with a past history of illicit substance and/or alcohol abuse (69.2% versus 31.2%, *χ* ^2^ = 4.144, *P* = 0.042); in female patients there were no significant differences (15.4% and 10.0%, resp.). The patients in the therapy-naïve group had on average significantly higher number of lymphocytes (10.0 ± 14.4 versus 1.51 ± 1.04 × 10^9^/L, *z* = −3.097, *P* = 0.002) and monocytes (3.5 ± 9.3 versus 0.38 ± 0.26×10^9^/L, *z* = −2.029, *P* = 0.042). The viral load and lymphocyte count correlated inversely and significantly in therapy-naïve patients (*n* = 33, rho = −0.464, *P* = 0.007), but not in patients with IFN-*α* treatment in the past. (*n* = 20, rho = −0.070 n.s.).

### 8.2. Depressive Symptoms

Overall, the mean baseline BDI score was 6.3 (95% CI = 4.7–8.6) with 16 patients (24.6%) having a score greater than 10 indicating the presence of depressive symptoms. In this subgroup with depressive symptoms, 10 patients (62.5%) claimed weight gain and only two patients (12.5%) claimed weight loss. In patients without depressive symptoms, only 4.1% reported weight gain.

On the other hand, according to the standardized psychiatric in-person interview, six patients met the DSM-IV criteria for a current major depression, single episode, mild or moderate. This group included five males and one female with a mean BDI score of 18.8 (SD = 4.5, range 13–26). These patients presented marked reduction in energy, interest, and pleasure and difficulty concentrating. These symptoms were associated with dysphoric mood and feelings of guilt and irritability. In general, these patients gained weight. They showed high* T*-scores not only on the BSI subscale “depression” (range 73–80) but also on the subscale “anger-hostility” (69–80) and “anxiety” (79–80). Three of the 6 patients were therapy-naïve.

The six patients with the diagnosis of minor depression were characterized by mild levels of depressed mood, difficulty concentrating, reduction in energy and/or interest, but not any reported sleep disturbances. The group included two males and four females, with a mean BDI score of 18.4 (SD = 7.0, range 11–32) and a mean BSI depression subscale score of 64.2 (SD = 4.1). Patients with minor depression were significantly younger as compared to the group with major depression (39.0 ± 13.2 versus 55.7 ± 7.4 years).

In the entire group of 12 patients with major/minor depression 66.6% were naïve to antiviral treatment and 42.6% experienced moderate to severe difficulty falling asleep and two patients reported hypersomnia. All indices from psychometric scales are summarized in Table 1 for depressed and nondepressed groups separately.

Compared with nondepressed patients, the group of patients with major/minor depression displayed significantly higher frequencies of thrombocytopenia and diabetes mellitus (Table 1). Depressed patients also tended to be more overweight. Age, gender distribution, and HCV mRNA levels were not different in the two groups, but leukocyte count was on average significantly higher in depressed patients (Table 1).

### 8.3. Fatigue Intensity and Interference Scores (BFI)

In the sample of 61 patients who completed the BFI, the global score ranged between 0 and 8 with a mean of 2.5 (SD = 2.1). All BFI measures for the entire group are shown in Table 1.

Twenty-three (37.7%) patients rated their fatigue at its worst in the severe range (≥7 on the 0–10 scale) and 13 (21.3%) patients in the moderate range (4–6 scores). Sixteen patients (26.2%) scored in the range between one and three and represented in the mild fatigue group. Fatigue was absent in nine (14.7%) patients.

Patients diagnosed with major/minor depression were more likely to rate their fatigue as severe compared with nondepressed patients (75% versus 28.6% *χ* ^2^ = 9.416, df = 1, *P* = 0.024).

**Figure 1:**
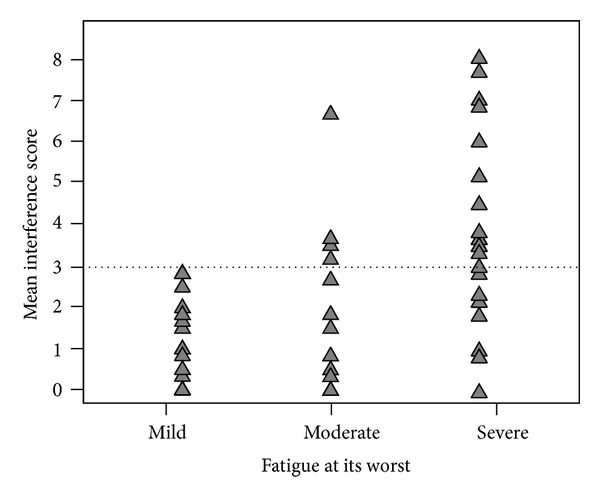
Interference of fatigue with mood and activity in patients categorized in mild, moderate, and severe fatigue intensity groups. Note: mean fatigue interference score = arithmetic mean of BFI items 4–9; the dotted line indicates the cutoff for moderate-to-severe interference score.

Figure 1 shows the individual interference score in patients categorized in mild, moderate, or severe fatigue intensity groups. The high variability in the feeling of fatigue as a contributor to functional limitations is notable. Of the 36 patients with moderate-to-severe fatigue intensity, only 18 rated their fatigue to have a marked impact on their daily activities and mood (interference score ≥3). Among these 18 patients, nine were diagnosed with major/minor depression and the other nine as nondepressed.

We noted that all patients without fatigue or fatigue related interference (*n* = 20) were nondepressed and infected only by genotype 1 virus. The frequency of past drug/alcohol use was higher in the group with fatigue interference score ≥3 relative to the “no fatigue” group with a trend for statistical significance (44.4% versus 27.7%; *χ* ^2^ = 3.633, df = 1, *P* = 0.057).

Comparing patients who were naïve to antiviral treatment and those with previous IFN-*α* treatment, we found a high frequency of treatment-naïve patients in the group with severe fatigue (69.6% versus 30.4%, *χ* ^2^ = 3.522, df = 1, *P* = 0.061).

### 8.4. Relationship between Psychometric Measures and Fatigue

**Figure 2:**
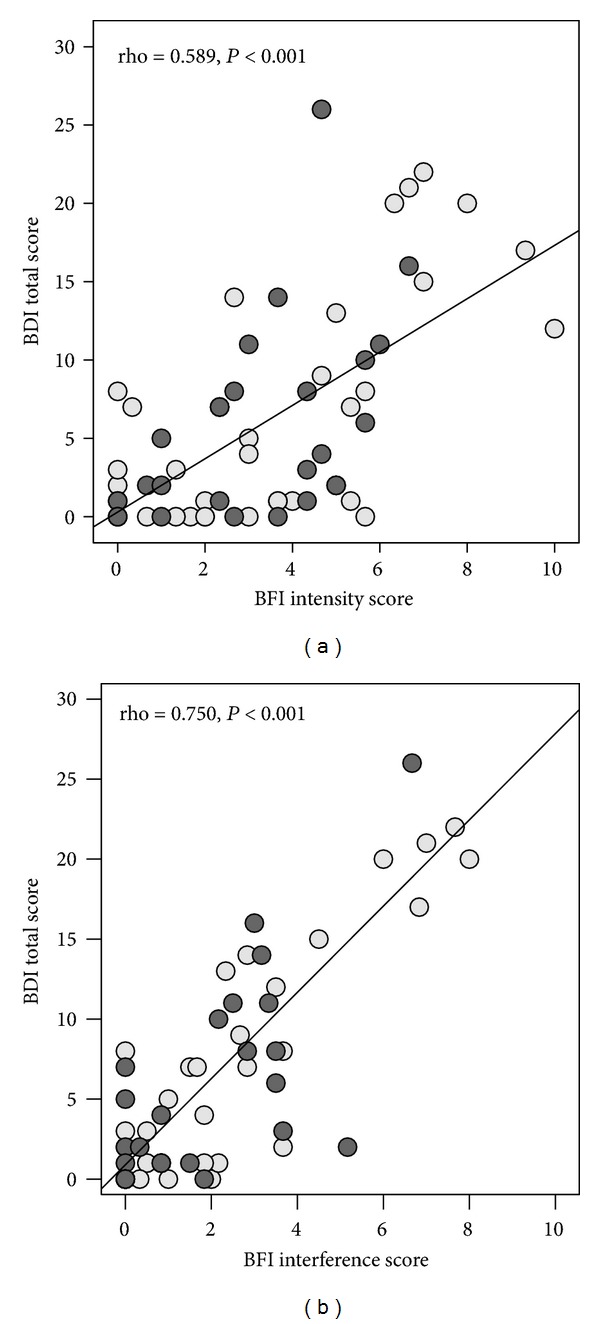
Relationship between BDI and BFI intensity scale (a) or BFI interference scale (b) in patients with chronic hepatitis C.* Note*. BDI: Beck Depression Inventory; BFI: Brief Fatigue Inventory; BFI intensity score = mean of three items (fatigue at present, at its usual level, and at its worst); ● previous IFN-*α* treatment; ○ treatment-naïve.

As expected, all psychometric scales correlated significantly with the BFI measure (data not shown). Most importantly, the relationship between psychometric measures and the BFI intensity score was weaker compared with the BFI interference score. As shown in Figure 2, the BDI total score correlated strongly with the BFI interference score (rho = 0.750, *P* < 0.001) but only moderately with the BFI intensity score (rho = 0.589, *P* < 0.001).

The Spearman correlation coefficient between the BFI interference score and the scores on the BSI subscale “anger-hostility” was high in patients with previous IFN-*α* treatment (*n* = 22, rho = 0.714, *P* < 0.001) but low in patients naïve to antiviral treatment (*n* = 32, rho = 0.411, *P* = 0.019).

### 8.5. Platelet 5HT Uptake Rate (SUR) and 5HT Content in HCV Patients

As shown in Tables 2 and 3, mean SUR and 5HT content were significantly higher in the entire group of patients in comparison to HCS, resulting in standardized effect size *d* of 0.586 and 0.362, respectively. These findings were based on the increased frequency of high or very high values in patients with 40% showing SURs above the 75th percentile of healthy control subjects (>63.5 pMol/10^9^ plts × 5 min). Similarly, 43.1% of the patients presented platelet 5HT content above the 75th percentile of HCSs (>488 ng/10^9^ plts.).

To confirm the finding of significant group differences, analysis was repeated by GLM. The main effect of “diagnostic group” (HCS and HCV patients) was significant for SUR (*F* = 15.368, df = 2, *P* < 0.001) after controlling for confounding variables. The putative confounders (age as covariate and gender, smoking, overweight, or hypertension as factors) did not seem to influence our result. With respect to the 5HT content, this model showed only a statistical trend for a significant main effect of diagnostic groups (*F* = 6.194, df = 2, *P* = 0.014). Of note, therapy-naïve patients (*n* = 39) displayed a significantly higher platelet 5HT content than the 26 patients with previous IFN-*α* treatment (medians: 488 versus 378 ng/10^9^ plts., resp., *z* = −2.229, *P* = 0.026).

In the next step, data for three groups: nondepressed patients (*n* = 49), patients with a BDI score >10 (*n* = 16), and patients with the diagnosis of major/minor depression (*n* = 12) were reanalysed separately. The mean SUR in nondepressed patients was slightly higher as compared to control subjects (Table 2). The two other subgroups displayed pronounced increased mean SURs versus HCS with high standardized effect sizes* d* in the range between 1.2 and 2.5. In accordance with high SUR in patients with major/minor depression, the platelet 5HT content was also significantly increased (Table 3).

Looking at only the 65 HCV patients, we found significant differences between nondepressed and depressed patients for SUR (*t*_63_ = −4.234, *P* < 0.001) and platelet-5HT content (*t*_62_ = −2.279, *P* = 0.040).

### 8.6. Impact of Thrombocytopenia on SERT Functionality

**Figure 3:**
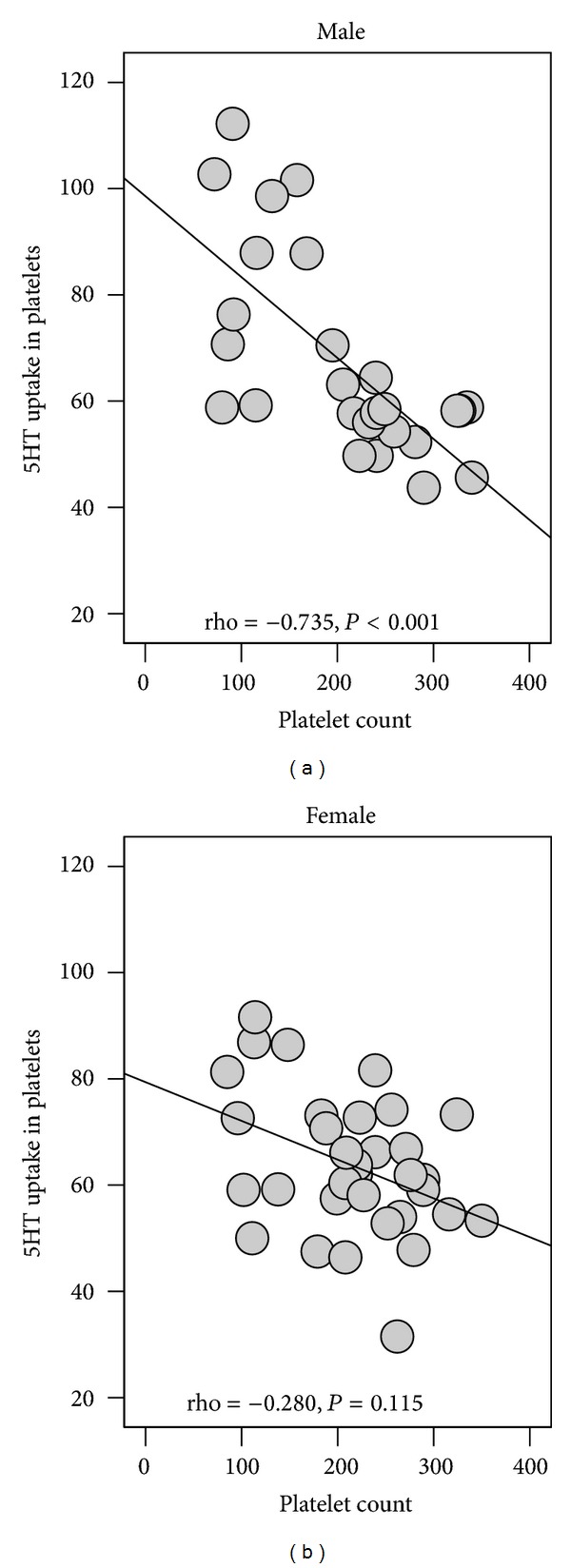
Relationship between blood platelet count and 5HT uptake rate in platelets of male and female patients with chronic hepatitis C. Note: 5HT uptake rate is expressed in pMol/10^9^ plts.×5 min and platelet count in ×10^9^ plts./L blood.

SUR, but not platelet-5HT level, inversely correlated with the platelet count (*n* = 61, rho = −0.528,  *P* < 0.001). Separate analysis by gender (Figure 3) revealed that this association existed only in males and was of strong value (rho = −0.739,  *P* < 0.001).

The mean SUR was significantly higher in patients with thrombocytopenia (*n* = 17) than in patients with normal platelet count (77.9 ± 17.7 versus 60.2 ± 11.7 pMol [^14^C]-5HT/10^9^ plts.×5 min, *t*_21.22_ = 3.855, *P* = 0.001). On the other hand, median 5HT content in platelets was not significantly different in both groups (419 versus 462 ng/10^9^ plts., resp.), however with a remarkable higher intersubject variability of values in the thrombocytopenia group (CV 53.1% versus 35.5%).

To address possible confounders related to thrombocytopenia, gender, and age, we reanalysed SUR in HCV patients with GLM. In this model, we found significant main effects of our variables in question “diagnostic group” (major/minor depression yes/no) (*F* = 4.792, *P* = 0.032) and “thrombocytopenia” (yes/no) (*F* = 16.422, *P* < 0.001). To exclude the contribution of low blood platelet count to the variability of the SUR, we reanalyzed data of patients with normal platelet count only (*n* = 46) and found again a significant main effect of “diagnostic group” (*F* = 24,815,  *P* < 0.001).

### 8.7. Relationship between Serotonergic Platelet Indices and Psychometric Measures

**Figure 4:**
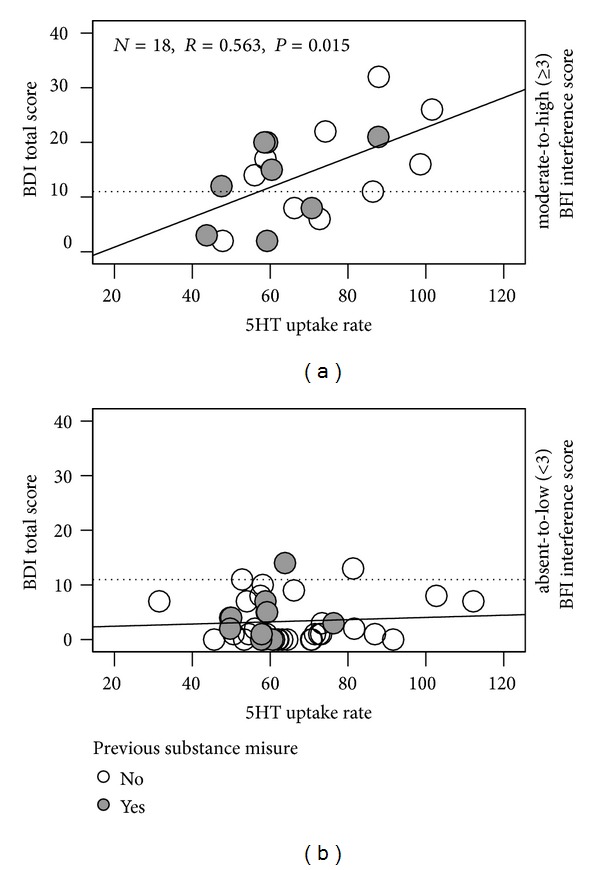
Relationship between the BDI total score and platelet 5HT uptake rate in patients with differed degrees of fatigue interference with mood and activity. Note: the dotted line indicates the cutoff for classification of BDI scores into depressed and nondepressed.

Whereas in the entire group of patients no such correlations were observed, we found a significant moderate correlation between the BDI total score and platelet 5HT uptake rate in the group of HCV patients with moderate-to-severe fatigue interference with mood and activity (Figure 4). To control for variables that could confound this relationship, we also performed partial correlation analysis. The relationship between BDI total score and platelet 5HT uptake (*r*_zero order_ = 0.563, *P* = 0.015) becomes stronger after controlling for age, gender, and thrombocytopenia (*r*_part._ = 0.710, *P* = 0.003).

### 8.8. Predictors of Depression in Untreated Patients with HCV

A summary of the results of cross-tabular univariate analysis of the relationship between the predictor variables and the likelihood of major/minor depression is shown in Table 4. The relative risk of depression was significantly increased in patients with moderate-to-severe fatigue interference with mood and activity, as well as in patients with thrombocytopenia or high platelet 5HT uptake rate. High body weight or high fatigue intensity scores, as well as gender or previous drug and/or alcohol use, were not significant predictors of depression.

## 9. Discussion

First, we found significant differences in mean SURs between HCS and the entire group of patients resulting in a medium effect size (Table 2). Looking at only the 16 patients with depressive symptoms (BDI score >10) or patients with the diagnosis of major/minor depression (*n* = 12), it was evident that these subgroups were characterized by significantly higher SUR. Notably, the SUR differences versus HCS were equivalent to large effect sizes (Table 2) supporting a possible role of the SERT in the expression of depressive symptoms in nontreated patients with CHC, although the pattern of findings is complex. The second main finding was the observation that especially in patients who perceived themselves as moderately to severely impaired by fatigue, the severity of depressive symptoms (expressed by the BD total score) was directly related to the SUR in platelets (Figure 4).

Since the investigation of SERT in the platelets of HCV patients is a new research area, there are no comparable studies in literature. On the other hand, psychiatric patients with major depression were frequently investigated for platelet SERT as an indirect index of central serotonergic function (assays of 5HT uptake kinetics in native platelets and crude platelet membrane binding studies with SERT-selective radioligands mostly with [^3^H]-imipramine or [^3^H]-paroxetine). Low SERT activity has been proposed as a trait marker for affective disorders [34]. Nevertheless, the findings are inconsistent; decreased, increased, and unchanged characteristics of the platelet SERT have been reported in depressed patients [10–13, 35]. So far, our finding of high SUR most pronounced in nontreated depressed patients with HCV is remarkable because recent* in vivo* neuroimaging studies in patients with major depression (drug-free for at least 3 weeks) showed significantly higher binding potentials of more specific SERT radioligands such as [^11^C]-DASB than the known nonselective [^125^I]-*β*-CIT in defined brain regions relative to healthy control subjects [36, 37].

Our finding of high platelet SUR in 40% of the HCV patients is in line with the hypothesis that one of the consequences of increased proinflammatory cytokine expression in patients with HCV is the upregulation of SERT-driven 5HT uptake in synaptic nerve endings and other peripheral cells. Proinflammatory cytokines seem to regulate SERT activity via activation of p38 mitogen-activated protein kinase (MAPK) [22]. Recently, Felger et al. (2011) investigated* in vivo* p38 MAPK response in peripheral blood lymphocytes to the initial injection of IFN-*α* in eleven patients with HCV: increase in the percentage of lymphocytes positive for phosphorylated p38 was significantly greater in patients who developed clinically significant symptoms of depression during the first 12 weeks of IFN-*α* treatment [38].

In the further discussion of our findings we abstained from speculating which mechanisms could underline the high SERT activity in some HCV patients. On the other hand, using available sample characteristic we found some indication that low blood platelet count could contribute to the high SER activity in platelets, at least in male patients (Figure 2). The prevalence and severity of thrombocytopenia are known to increase with increasing hepatocellular damage in chronic HCV [39]. Thus, we should take into account that this haematological abnormality could influence platelet-related assessments and thus obscure the interpretation of experimental findings.

In the univariate analysis, both conditions (thrombocytopenia and high SUR) were associated with significantly increased relative risk for depression (Table 4). Nevertheless, high platelet SERT activity seems not to be just a simple biomarker for the ubiquitous symptom “depression” which is frequently assumed to be present in a large number of patients with CHC. Further available sample characteristics in terms of fatigue and depressive symptom measures indicated a rather complex picture of interactions with SUR.

In general, fatigue and axis I psychiatric disorders, particularly depression, are thought to be often associated [40]. To our knowledge, only two studies with HCV patients examined this association in more detail [4, 8]. Despite this research, the nature of this association in CHC remains only partially understood. Extending these two previous investigations, our data analysis showed that significant levels of fatigue occur independently from current psychiatric disorder. About 59% of the patients rated their fatigue intensity as moderate-to-severe, but only 18.4% were diagnosed as having major/minor depression. For comparison, Dwight et al. [4] found a 28% prevalence of major depression among 50 patients with HCV (and lifetime diagnosis of drug abuse/dependence in 46%). They were evaluated with structured psychiatric interviews by telephone and standardized self-rating instruments. The reported mean BDI score of 12.3 (95% CI 3.6–21) for this subgroup of 14 patients (with two patients on IFN-*α* treatment) was surprisingly low. In our study the 12 patients diagnosed with major/minor depression displayed a mean BDI sore of 18.6 (95% CI 15–22).

We do not know whether or not the timing of fatigue antecedes depression in patients with chronic HCV but prefer the assumption that the presence of fatigue may lead to depression rather than occurring as its consequence. From our results, we would conclude that it is not the fatigue per se, but the subjective perception of fatigue as an impairment factor with high distress potential that could contribute to the development of clinically relevant symptoms of depression. Some patients with chronic HCV may experience their fatigue as distressing, but others with the same fatigue intensity would not. For a better understanding of the relationship between depression and fatigue in HCV patients, this difference should be explored in more detail. Stress may additionally affect immune function through activation of the HPA axis and the sympathetic nervous system [41, 42]. Moreover, chronic stress may contribute to neuropsychiatric manifestations such as anxiety, depression, and cognitive dysfunction [42]. Our finding of significantly higher mean leukocyte count, specifically in patients with major/minor depression, could be stress related. Patients diagnosed with major/minor depression experienced significantly higher psychological distress relative to nondepressed patients (Table 1).

In this context, it is interesting that the fatigue level was much lower associated with different psychometric measures than the fatigue interference score (Figure 2). In a study by Kramer et al. (2005), 120 untreated chronic HCV patients without a current or past history of psychiatric disorders had a BFI total mean score ranging between 0.8 and 10 [31]. The authors reported that 51% of the patients claimed to be affected by severe fatigue. Also, these findings underline that significant levels of fatigue occur in some HCV patients independently from a psychiatric disorder.

Lastly, of high interest is the group of 18 patients who perceived themselves as moderately to severely impaired by fatigue (BFI interference scale ≥3). The direct and significant relationship between BDI score and SUR, specifically in this subgroup of patients (Figure 4), allows us to assume that the two markers, high fatigue interference score and high SUR, both increase the likelihood of clinically significant symptoms of depression over that of either marker alone.

On the other hand, some literature also points to a relationship between fatigue and different aspects of behaviour and communication problems in patients with HCV [8, 43]. Similar to findings reported by McDonald et al. [8], we noted a significant association between scores on the fatigue interference scale and the BSI dimension “hostility-anger.” More important for clinical practice, however, could be our observation that the strength of this association was different between treatment-naïve patients (low) and patients with previous IFN-*α* based treatment (strong).

We noted that the platelet 5HT content in HCV patients was highly variable and also remained variable in the group with minor/major depression. The explanation for this observation is not simple. The synthesis and release of 5HT from the gastrointestinal tract into the portal circulation with the subsequent degradation of 5HT via monoamine oxidase A (MAO-A) in the liver could have some impact on this variability. As MAO-A and MAO-B are mitochondrial enzymes, serotonin needs to be transported into the hepatocytes and/or stellate cells. The demonstration of SERT in stellate cells suggests its potential role in the degradation of 5HT beside the hepatocytes which are known to contain both MAO-A and MAO-B [44, 45].

The present study has some limitations. First, our sample size is fairly modest, although it is larger than the previously reported study of Dwight et al. [4] that investigated the relationship between depression and fatigue measures. The patients included were not all naïve to antiviral treatment. Some sample characteristics indicate that a separate consideration of treatment-naïve and those with previous IFN-*α* based therapy could be necessary. For example, we noted significantly lower mean 5HT content in patients with IFN-*α* treatment in the past relative to the group of treatment-naïve patients; lymphocyte and monocyte counts were on average higher in treatment-naïve patients as well. Second, we have not assessed other relevant characteristics of the 5HT uptake in platelets, such as maximum uptake rate (*V*_max⁡_) and Michaelis constant (*K*_*m*_). Therefore we cannot say whether the measured high 5HT uptake rate in some patients is due to an increased number of SERT in the platelet membrane (*V*_max⁡_) or due to a better affinity of the given SERT for 5HT (*K*_*m*_), or both.

The relative modest sample size of HCV patients and the further splitting of these patients in defined subgroups might have limited statistical validity. One can assume that positive findings in these subgroups are due to some bias or outliers. However, outliers were removed from the data set; we recognized the presence of thrombocytopenia in some HCV patients as an additional factor contributing to increased SUR in platelets. A separate consideration of depressed patients with thrombocytopenia and those without thrombocytopenia could be necessary. Finally, patients with known HCV infection attending specialist centres and selected according to our inclusion criteria for the study could represent a special group of HCV patients and thus limit the generalisation of our findings to other populations.

## 10. Conclusion 

Patient's reports of the extent to which fatigue interferes with different aspects of their daily lives could be a practicable measure, differentiating CHC patients with and without a risk of depression. In our sample, the relative risk for developing mild-to-moderate symptoms of depression was about 7 times higher in patients who perceived themselves as moderately to severely impaired by fatigue (BFI interference scale ≥3). Ongoing high SERT activity, having the potential to diminish extracellular synaptic 5HT level, may act as a trigger for the onset of clinically significant depression specifically in patients experiencing fatigue as distressing and impairing. Given the preliminary nature of this study, further research is needed to replicate our findings. Based on the observed effect size *d* in our sample for SUR, we can expect that the likely size of the effect in larger population of nontreated HCV patients with fatigue and depressive symptoms and without previous pharmacological treatment (antidepressants) maybe also high. By contrast, platelet 5HT content does not seem to be a perfect biomarker for depression in nontreated patients with HCV.

## Figures and Tables

**Table 1:** Demographic and clinical characteristics of patients with chronic HCV.

	All patients *n* = 65	Major/minor depression *n* = 12	Nondepressed *n* = 53	*χ* ^2^ test Mann-Whitney *U*-test depressed versus nondepressed
Male/female	29/36	7/5	22/31	

Age (years)	46.2 ± 11.9	48.3 ± 11.7	46.1 ± 11.6	

BMI (kg/m²)	24.8 ± 3.3	26.2 ± 4.5	24.8 ± 3.3	*χ* ^2^ = 3.340, *P* = 0.093
Overweight (>25), % (*n*)	44.6% (29)	**66.7% (8)**	39.6 (21)	

Smokers, % (*n*)	42.8% (26)	50% (6)	41.5% (22)	

HCV mRNA ( log IU/mL)	6.25 ± 0.59	6.24 ± 0.51	6.25 ± 0.62	
Low <600 000 IU/mL, % (*n*)	24.6% (15/61)	16.7% (2)	26.5% (13/49)	
Genotype 1, %	84.4%	75%	86.5%	
Genotypes 2, 3, or 4, %	15.6%	25%	13.5%	

Leukocyte count (×10^9^/L)	5.95 ± 1.79	7.15 ± 2.21	5.69 ± 1.60	*Z* = −2.082, *P* = 0.037
	*n* = 61	*n* = 11	*N* = 50	

Platelet count (×10^9^/L)	209 ± 76	152 ± 62	223 ± 73	*z* = −2.821, *P* = 0.006
Thrombocytopenia, % (*n*)	26.2% (17)	58.3% (7)	18.5% (10**)**	*χ* ^2^ = 8.139, *P* = 0.004

Hypertonia, % (*n*)	29.2% (19)	33.3% (4)	28.3% (15)	

Diabetes mellitus, % (*n*)	7.7% (5)	**25.0%** (3)	3.8% (2)	*χ* ^2^ = 6.209, *P* = 0.013

Therapy-naïve, % (*n*)	60% (39)	66.7% (8)	58.5% (31)	

Past drug/alcohol use, % (*n*)	27.7% (19)	33.3% (4)	28.3% (15)	

Past episodes of depressive symptoms, % (*n*)	16.9% (11)	**33.3%** (4)	13.2% (7)	*χ* ^2^ = −2.819, *P* = 0.093

Beck Depression Inventory				
Total score	6.3 ± 7.6	18.3 ± 6.6	3.6 ± 4.1	All measures significantly different at the *P* < 0.001 level
Emotional/cognitive	3.0 ± 4.8	11.1 ± 5.6	1.3 ± 2.4	
Somatic/vegetative	3.1 ± 3.2	7.2 ± 2.3	2.3 ± 2.3	

Brief Symptom Inventory				
Depression	51.3 ± 12.4	70.2 ± 9.4	46.6 ± 7.6	All measures significantly different at the *P* < 0.001 level
Anxiety	50.7 ± 13.3	66.5 ± 11.2	46.8 ± 10.7	
Anger-Hostility	55.6 ± 12.2	68.4 ± 10.0	52.4 ± 10.6	
Global severity index	47.0 ± 17.3	70.8 ± 11.8	41.7 ± 13.3	

Brief Fatigue Inventory				
Total mean	2.5 ± 2.1	5.4 ± 1.9	1.9 ± 1.6	Three measures significantly different at the *P* < 0.001 level *z* = −2.248, *P* = 0.025
Interference score	2.1 ± 2.2	5.1 ± 2.2	1.4 ± 1.1	
Intensity score (3 items)	3.4 ± 2.5	5.9 ± 2.0	2.8 ± 2.2	
fatigue at its worst	4.6 ± 3.1	6.5 ± 2.6	4.1 ± 3.1	

Note: values for continuous variables are expressed as mean ± SD.

**Table 2:** Comparison of 5HT uptake rate (SUR) in healthy control subjects and patients with chronic HCV.

	*N*	Mean (SD)	95% CI (mean)	*t*-test versus control group	Effect size (*d*)	95% CI (*d*)
Control group	65	**57.3 ( 9.4)**	**54.9–59.6**			
Patients total	65	64.8 (15.5)	61.0–68.7	*t*_105.5_ = 3.345, *P* = 0.001	0.585	0.23–0.93
Patients (BDI ≤ 10)	49	62.5 (14.6)	58.5–66.9	*t*_77.34_ = 2.272, *P* = 0.026	0.437	0.061–0.812
Patients (BDI > 10)	16	**71.3 (17.0)**	**62.2–80.4**	*t*_17.32_ = 3.172, *P* = 0.005	1.245	0.665–1.825
Patients with major/minor depression	12	**80.1 (16.9)**	**63.3–90.8**	*t*_12.3_ = 4.545, *P* = 0.001	2.105	1.40–2.80

Note: the standardized mean-difference effect size (*d*) was computed from means and standard deviations; SUR is expressed in pMol/10^9^ plts. × 5 min.

**Table 3:** Comparison of serotonin levels in platelets of healthy control subjects and patients with chronic HCV.

	*n*	Mean (SD)	95% CI (mean)	*t*-test versus control group	Effect size (*d*)	95% CI (*d*)
Control group	65	**412 (125)**	**381–443**			
Patients total	64	470 (189)	423–518	*t*_108.92_ = 2.050, *P* = 0.043	0.362	0.014–0.709
Patients (BDI ≤ 10)	49	453 (187)	399–507	n.s. (*P* = 0.187)	0.240	−0.052–0.533
Patients (BDI > 10)	15*	**585 (302)**	**424–746**	*t*_16.79_ = 2.164, *P* = 0.045	1.012	0.429–1.595
Patients with major/minor depression	11*	**608 (228)**	**435–709**	*t*_11.34_ = 2.585, *P* = 0.025	1.367	0.693–2.043

Note: the standardized mean-difference effect size (*d*) was computed from means and standard deviations; platelet-5HT levels are expressed in ng/10^9^ plts.

*Without the extremely high value (outlier) of 1425 ng/10^9^ plts.

**Table 4:** Chi-Square Test of association between the predictor variables and the likelihood of depression.

Predictor variables	Total	Depression	*χ* ^2^/*P*	Relative risk (95% CI)
Overweight				
BMI > 25 kg/m^2^	29	8 (27.6%)	2.896, *P* = 0.089	2.483 (0.830–7.429)
BMI ≤ 25 kg/m^2^	36	4 (11.1%)		

Thrombocytopenia				
Yes	17	7 **(41.2%)**	**7.891, ** ***P*** = **0.005**	**3.953 (1.446–10.805)**
No	48	5 (10.4%)		

BFI interference score				
≥3 (moderate to severe)	18	9 **(50.0%)**	**14.863, ** ***P*** < **0.001**	**7.167 (2.191–23.443)**
<3 (absent to mild)	43	3 (7.0%)		

BFI intensity score				
7–10 (moderate to severe)	35	9 (25.7%)	3.056, *P* = 0.080	3.214 (0.759–13.616)
0–6 (no to mild)	25	2 (8.0%)		

5HT uptake groups				
>75th percentile	26	9 **(34.6%)**	**7.512, ** ***P*** = **0.006**	**4.50 (1.344–15.072)**
≤75th percentile	39	3 (7.7%)		

5HT content groups				
>75th percentile	28	7 (25.0%)	1.397, *P* = 0.237	1.850 (0.656–5.220)
≤75th percentile	37	5 (13.5%)		

## References

[1] Johnson ME, Fisher DG, Fenaughty A, Theno SA (1998). Hepatitis C virus and depression in drug users. *American Journal of Gastroenterology*.

[2] Dantzer R, O’Connor JC, Freund GG, Johnson RW, Kelley KW (2008). From inflammation to sickness and depression: when the immune system subjugates the brain. *Nature Reviews Neuroscience*.

[3] Leutscher PDC, Lagging M, Buhl MR (2010). Evaluation of depression as a risk factor for treatment failure in chronic hepatitis C. *Hepatology*.

[4] Dwight MM, Kowdley KV, Russo JE, Ciechanowski PS, Larson AM, Katon WJ (2000). Depression, fatigue, and functional disability in patients with chronic hepatitis C. *Journal of Psychosomatic Research*.

[5] Kraus MR, Schafer A, Csef H, Scheurlen M, Faller H (2000). Emotional state, coping styles, and somatic variables in patients with chronic hepatitis C. *Psychosomatics*.

[6] Hunt CM, Dominitz JA, Bute BP, Waters B, Blasi U, Williams DM (1997). Effect of interferon-*α* treatment of chronic hepatitis C on health- related quality of life. *Digestive Diseases and Sciences*.

[7] Poynard T, Cacoub P, Ratziu V (2002). Fatigue in patients with chronic hepatitis C. *Journal of Viral Hepatitis*.

[8] McDonald J, Jayasuriya R, Bindley P, Gonsalvez C, Gluseska S (2002). Fatigue and psychological disorders in chronic hepatitis C. *Journal of Gastroenterology and Hepatology*.

[9] Mück-Šeler D, Jakovljević M, Pivac N (1996). Platelet 5-HT concentrations and suicidal behaviour in recurrent major depression. *Journal of Affective Disorders*.

[10] Neuger J, El Khoury A, Kjellman BF, Wahlund B, Åberg-Wistedt A, Stain-Malmgren R (1999). Platelet serotonin functions in untreated major depression. *Psychiatry Research*.

[11] Hrdina PD, Bakish D, Ravindran A, Chudzik J, Cavazzoni P, Lapierre YD (1997). Platelet serotonergic indices in major depression: up-regulation of 5-HT(2A) receptors unchanged by antidepressant treatment. *Psychiatry Research*.

[12] Franke L, Schewe H-J, Müller B (2000). Serotonergic platelet variables in unmedicated patients suffering from major depression and healthy subjects: relationship between 5HT content and 5HT uptake. *Life Sciences*.

[13] Franke L, Schewe H-J, Uebelhack R, Müller-Oerlinghausen B (2003). High platelet-serotonin uptake activity is associated with a rapid response in depressed patients treated with amitriptyline. *Neuroscience Letters*.

[14] Bonaccorso S, Marino V, Puzella A (2002). Increased depressive ratings in patients with hepatitis C receiving interferon-*α*-based immunotherapy are related to interferon-*α*-induced changes in the serotonergic system. *Journal of Clinical Psychopharmacology*.

[15] Russo S, Kema IP, Haagsma EB (2005). Irritability rather than depression during interferon treatment is linked to increased tryptophan catabolism. *Psychosomatic Medicine*.

[16] Fontana RJ, Kronfol Z, Lindsay KL (2008). Changes in mood states and biomarkers during peginterferon and ribavirin treatment of chronic hepatitis C. *American Journal of Gastroenterology*.

[17] Schäfer A, Scheurlen M, Seufert J (2010). Platelet serotonin (5-HT) levels in interferon-treated patients with hepatitis C and its possible association with interferon-induced depression. *Journal of Hepatology*.

[18] Kronfol Z, Litman HJ, Back-Madruga C (2011). No increase in depression with low-dose maintenance peginterferon in prior non-responders with chronic hepatitis C. *Journal of Affective Disorders*.

[19] Torres GE, Gainetdinov RR, Caron MG (2003). Plasma membrane monoamine transporters: structure, regulation and function. *Nature Reviews Neuroscience*.

[20] Mössner R, Heils A, Stöber G, Okladnova O, Daniel S, Lesch K-P (1998). Enhancement of serotonin transporter function by tumor necrosis factor alpha but not by interleukin-6. *Neurochemistry International*.

[21] Zhu C-B, Blakely RD, Hewlett WA (2006). The proinflammatory cytokines interleukin-1beta and tumor necrosis factor-alpha activate serotonin transporters. *Neuropsychopharmacology*.

[22] Tsao C-W, Lin Y-S, Cheng J-T (2008). Interferon-*α*-induced serotonin uptake in Jurkat T cells via mitogen-activated protein kinase and transcriptional regulation of the serotonin transporter. *Journal of Psychopharmacology*.

[23] Mössner R, Daniel S, Schmitt A, Albert D, Lesch K-P (2001). Modulation of serotonin transporter function by interleukin-4. *Life Sciences*.

[24] Sheehan DV, Lecrubier Y, Sheehan KH (1998). The Mini-International Neuropsychiatric Interview (M.I.N.I.): the development and validation of a structured diagnostic psychiatric interview for DSM-IV and ICD-10. *Journal of Clinical Psychiatry*.

[25] Holtzheimer PE, Veitengruber J, Wang CC (2010). Utility of the Beck Depression Inventory to screen for and track depression in injection drug users seeking hepatitis C treatment. *General Hospital Psychiatry*.

[26] Golden J, Conroy RM, O’Dwyer AM (2007). Reliability and validity of the Hospital Anxiety and Depression Scale and the Beck Depression Inventory (Full and FastScreen scales) in detecting depression in persons with hepatitis C. *Journal of Affective Disorders*.

[27] Dieperink E, Ho SB, Thuras P, Willenbring ML (2003). A prospective study of neuropsychiatric symptoms associated with interferon-*α*-2b and ribavirin therapy for patients with chronic hepatitis C. *Psychosomatics*.

[28] Mendoza TR, Wang XS, Cleeland CS (1999). The rapid assessment of fatigue severity in cancer patients: use of the brief fatigue inventory. *Cancer*.

[29] Mendoza TR, Laudico AV, Wang XS (2010). Assessment of fatigue in cancer patients and community dwellers: validation study of the filipino version of the brief fatigue inventory. *Oncology*.

[30] Anderson KO, Getto CJ, Mendoza TR (2003). Fatigue and sleep disturbance in patients with cancer, patients with clinical depression, and community-dwelling adults. *Journal of Pain and Symptom Management*.

[31] Kramer L, Hofer H, Bauer E (2005). Relative impact of fatigue and subclinical cognitive brain dysfunction on health-related quality of life in chronic hepatitis C infection. *AIDS*.

[32] Huang T-W, Lin C-C (2009). The mediating effects of depression on sleep disturbance and fatigue: symptom clusters in patients with hepatocellular carcinoma. *Cancer Nursing*.

[33] Franke L, Schmidtmann M, Riedl A, Van Der Voort I, Uebelhack R, Mönnikes H (2010). Serotonin transporter activity and serotonin concentration in platelets of patients with irritable bowel syndrome: effect of gender. *Journal of Gastroenterology*.

[34] Jans LAW, Riedel WJ, Markus CR, Blokland A (2007). Serotonergic vulnerability and depression: assumptions, experimental evidence and implications. *Molecular Psychiatry*.

[35] Yubero-Lahoz S, Robledo P, Farré M, de la Torre R (2013). Platelet SERT as a peripheral biomarker of serotonergic neurotransmission in the central nervous system. *Current Medicinal Chemistry*.

[36] Meyer JH, Houle S, Sagrati S (2004). Brain serotonin transporter binding potential measured with carbon 11-labeled DASB positron emission tomography: effects of major depressive episodes and severity of dysfunctional attitudes. *Archives of General Psychiatry*.

[37] Cannon DM, Ichise M, Rollis D (2007). Elevated Serotonin Transporter Binding in Major Depressive Disorder Assessed Using Positron Emission Tomography and [11C]DASB; Comparison with Bipolar Disorder. *Biological Psychiatry*.

[38] Felger JC, Alagbe O, Pace TWW (2011). Early activation of p38 mitogen activated protein kinase is associated with interferon-alpha-induced depression and fatigue. *Brain, Behavior, and Immunity*.

[39] Weksler BB (2007). Review article: the pathophysiology of thrombocytopenia in hepatitis C virus infection and chronic liver disease. *Alimentary Pharmacology and Therapeutics*.

[40] Arnold LM (2008). Understanding fatigue in major depressive disorder and other medical disorders. *Psychosomatics*.

[41] Webster Marketon JI, Glaser R (2008). Stress hormones and immune function. *Cellular Immunology*.

[42] Chrousos GP (2009). Stress and disorders of the stress system. *Nature Reviews Endocrinology*.

[43] Obhrai J, Hall Y, Anand BS (2001). Assessment of fatigue and psychologic disturbances in patients with hepatitis c virus infection. *Journal of Clinical Gastroenterology*.

[44] Ruddell RG, Oakley F, Hussain Z (2006). A role for serotonin (5-HT) in hepatic stellate cell function and liver fibrosis. *American Journal of Pathology*.

[45] Billett EE (2004). Monoamine Oxidase (MAO) in Human Peripheral Tissues. *NeuroToxicology*.

